# Hand, Foot, and Mouth Disease Surveillance in Anhui, After Introduction of the Enterovirus 71 Vaccine and During the COVID-19 Pandemic: Ecological Interrupted Time Series Study

**DOI:** 10.2196/99663

**Published:** 2026-09-24

**Authors:** Kangguo Li, Jiadong Wu, Wanwan Ma, Tao Chen, Yunzhi Zenghuang, Jia Rui, Zeyu Zhao, Jiabing Wu, Tianmu Chen

**Affiliations:** 1 State Key Laboratory of Molecular Vaccinology and Molecular Diagnostics, School of Public Health Xiamen University Xiamen, Fujian China; 2 Fuzhou Center for Disease Control and Prevention Fuzhou, Fujian China; 3 Department of Acute Infectious Disease Prevention and Control Anhui Provincial Center for Disease Control and Prevention Hefei, Anhui China; 4 Department of Epidemiology and Health Statistics, Xiangya School of Public Health Central South University Changsha, Hunan China; 5 WorldPop, School of Geography and Environmental Science University of Southampton Southampton, England United Kingdom

**Keywords:** hand, foot, and mouth disease, enterovirus A71, enterovirus 71 vaccine, coxsackievirus A16, disease surveillance, interrupted time series analysis, COVID-19, spatiotemporal analysis, China, public health surveillance

## Abstract

**Background:**

Routine surveillance is important for understanding hand, foot, and mouth disease (HFMD) after monovalent enterovirus 71 (EV71) vaccine introduction, but interpretation is difficult when etiologic typing is sparse and the COVID-19 pandemic overlaps with the postvaccine era.

**Objective:**

This study described surveillance-derived changes in HFMD burden and typed serotype composition in Anhui Province, China, after EV71 vaccine introduction and during the COVID-19 pandemic.

**Methods:**

We conducted a retrospective ecological surveillance study using HFMD records from January 2008 to December 2023. The primary analysis used negative-binomial interrupted time series (ITS) counterfactual models for all reported HFMD cases and typed EV71 monthly counts. Vaccine-era models were trained using data from January 2013 through May 2016; the previous specification covering January 2010 through May 2016 was retained as a sensitivity analysis. COVID-19–period models were trained using data from January 2008 through December 2019. Secondary analyses assessed spatial, temporal, and age patterns. Vaccine coverage histories, individual vaccination records, specimen submission metadata, and laboratory platform-change metadata were unavailable; therefore, models estimated calendar-based surveillance deviations rather than vaccine effectiveness.

**Results:**

Anhui reported 1,292,579 HFMD cases from 2008 to 2023, including 47,990 (3.7%) typed cases. EV71 declined from 45.8% (8479/18,534) of typed cases before vaccine introduction to 3.4% (578/16,930) during COVID-19, while other enteroviruses increased from 29.6% (5487/18,534) to 81.8% (13,848/16,930). There were 123 deaths from 2008 to 2023, corresponding to 9.52 deaths per 100,000 reported cases; no deaths were recorded from 2020 to 2023. In primary vaccine-era ITS models, typed EV71 counts remained below the predicted trajectory in 2017 to 2019: 470 observed vs 659 predicted cases (95% prediction interval [PI] 467-933) in 2017, 181 vs 531 (95% PI 379-798) in 2018, and 63 vs 427 (95% PI 305-662) in 2019. All reported HFMD cases were not uniformly below the vaccine-era counterfactual. During the COVID-19 pandemic, all reported HFMD cases remained below prediction through 2023, with the largest relative difference in 2022 (28,385 observed vs 175,601 predicted; 95% PI 111,465-268,202). The proportion of reported children aged ≥4 years increased from 19% (50,503/266,132) during 2017 to 2019 to 29.1% (44,133/151,795) during 2021 to 2023 (odds ratio 1.75, 95% CI 1.72-1.78; *P*<.001).

**Conclusions:**

Typed EV71 surveillance signals declined after EV71 vaccine introduction, and overall HFMD burden was strongly disrupted during the COVID-19 pandemic. However, sparse typing, unavailable vaccination and testing policy metadata, and the aggregated other enteroviruses category limit causal interpretation. These findings should be read as surveillance signals, not as direct estimates of population-level serotype replacement or individual-level vaccine protection.

## Introduction

Hand, foot, and mouth disease (HFMD) remains an important childhood infection in China and is caused by multiple enteroviruses [1,2]. Enterovirus 71 (EV71) has long been of particular concern because it is disproportionately associated with severe neurological complications and death [3-5]. Since inactivated monovalent EV71 vaccines were introduced in China in 2016, postlicensure surveillance has been used to examine whether typed HFMD patterns have shifted away from EV71 and toward non-EV71 enteroviruses [6-9].

Routine surveillance is essential for detecting population-level signals, but it is not equivalent to an individually linked vaccine effectiveness study [10]. HFMD surveillance systems do not type every reported case, and the typed subset may be affected by case selection, disease severity, reagent availability, local laboratory capacity, and changes in diagnostic platforms [11,12]. These constraints are especially important when broad categories such as “other enteroviruses” are used because changes in testing breadth may alter which infections become visible to the surveillance system [12-16].

Interpretation became more difficult after January 2020, when the COVID-19 pandemic altered health care use, child contact patterns, and disease reporting [17-23]. Intensive nonpharmaceutical interventions (NPIs) coincided with sharp short-term reductions in HFMD in multiple settings [17-20], while the later rebound has been interpreted as compatible with renewed transmission and an expanded susceptible pool [21-23]. In practice, EV71 vaccine introduction and the COVID-19 period are temporally contiguous disruptions in the same surveillance series. For an ecological dataset, this overlap limits the clean separation of vaccine-era trend shifts from pandemic-era perturbation.

Many province-level HFMD reports have described declining EV71 detection and rising non-EV71 detection after vaccine introduction [6,19,24-26]. What is often less explicit is the inferential boundary: with sparse typing and incomplete testing metadata, typed serotype composition is a surveillance signal rather than a direct estimate of population serotype structure [27]. Long-term provincial surveillance remains valuable because it can show how routine reporting, etiologic typing, seasonality, spatial heterogeneity, severe outcomes, and pandemic-era rebound evolved in the same public health system.

Using provincial HFMD surveillance data from Anhui Province from 2008 to 2023, we aimed to describe how reported burden and typed serotype composition changed after EV71 vaccine introduction and during the COVID-19 period. We prioritized ecological interrupted time series (ITS) counterfactual analyses of all reported HFMD cases and typed EV71 cases, while treating spatial analyses, county-level typed-dominance summaries, seasonality assessment, and joinpoint regression as secondary descriptive analyses. Because individual vaccination records and province-wide vaccine coverage histories were unavailable, the study was framed as an ecological surveillance analysis rather than a causal assessment of vaccine effectiveness.

## Methods

### Study Design and Data Source

We conducted a retrospective ecological surveillance study using deidentified HFMD records provided by the Anhui Provincial Center for Disease Control and Prevention. The analytic window was January 1, 2008, through December 31, 2023. Anhui Province comprises 16 prefecture-level cities and 104 county-level administrative units. Because different surveillance outputs were available at different spatial resolutions, analyses were conducted at mixed levels by design: prefecture-level city units were used for incidence mapping and spatiotemporal cluster detection; county-level units were used for typed-dominance mapping; and province-level aggregation was used for seasonality, joinpoint, and ITS analyses.

The surveillance extract included date of symptom onset, administrative unit, age, sex, clinical severity, and etiologic typing result when available. Population denominators were obtained from the Anhui Statistical Yearbook [28]. HFMD cases were reported through the statutory notifiable disease surveillance system. Clinical diagnosis followed the national HFMD diagnostic standard, which requires compatible symptoms, such as fever with rash or vesicles affecting the hands, feet, mouth, or buttocks; cases without rash are not clinically diagnosed as HFMD [29]. We followed the STROBE (Strengthening the Reporting of Observational Studies in Epidemiology) reporting principles, where applicable, to an aggregated surveillance analysis [30].

### Surveillance and Testing Context

Under the national diagnostic standard, severe HFMD includes central nervous system involvement, such as poor mental status, somnolence, startle, delirium, headache, vomiting, limb tremor, myoclonus, nystagmus, ataxia, weakness, acute flaccid paralysis, convulsions, or meningeal signs; critical disease includes severe central nervous system injury, respiratory dysfunction, or circulatory dysfunction [29]. In this analysis, severe cases were records marked as severe in the surveillance extract. The extract contained a death-date field but did not contain follow-up information on neurological sequelae.

Several surveillance features define the boundary of interpretation. June 1, 2016, was used as the EV71 vaccine–era boundary, and January 1, 2020, to December 31, 2023, was treated as the COVID-19 period. Etiologic typing was incomplete and time varying: 47,990 (3.7%) of 1,292,579 reported cases had a recorded etiologic result, with annual typing fractions ranging from 0.3% (76/26,359) in 2008 to 8.4% (4429/52,712) in 2020. Typed analyses therefore describe surveillance-derived typed composition rather than population serotype structure. The surveillance extracts also lacked individual vaccination records, province-wide vaccine coverage histories, annual specimen submission criteria, and laboratory platform-change records. These gaps precluded direct estimation of vaccine effectiveness, adjustment for testing policy changes, or attribution of changes in the aggregated other enteroviruses category to true population-level serotype replacement.

### Outcomes and Definitions

The primary burden end point was the monthly number of reported HFMD cases. The primary typing end point was the monthly number of typed EV71 cases. Typed cases were also categorized as coxsackievirus A16 (CV-A16) or other enteroviruses. In this surveillance extract, other enteroviruses represented typed results recorded as neither EV71 nor CV-A16; the category could not distinguish coxsackievirus A6 (CV-A6), coxsackievirus A10 (CV-A10), or other individual serotypes. Deaths were identified from nonmissing death dates, and case fatality was summarized as deaths per 100,000 reported cases.

For descriptive analyses, we summarized annual reported cases, annual incidence, typing fractions, typed serotype composition, sex distribution, age distribution, severe cases, deaths, and case fatality. Age-shift analyses grouped cases with known age as children aged ≤1 year, aged 2 to 3 years, or aged ≥4 years. For county-level typed-dominance maps, we masked county-year observations with <10 typed cases because dominant-serotype assignments based on small number of typed cases are not epidemiologically stable.

### Statistical Analysis

We first described overall burden, typed composition, and age-sex structure over time. Seasonality was summarized with epidemic curves and a wavelet analysis of weekly reported cases. Annual incidence maps at the prefecture-level city scale, spatiotemporal scan statistics, Moran *I*, and joinpoint regression were retained as secondary descriptive analyses. Retrospective spatiotemporal scan statistics used a discrete Poisson model, a maximum spatial cluster size of 15% of the population at risk, a maximum temporal window of 1 year, and monthly temporal aggregation. Cluster-size sensitivity analyses used maximum spatial windows of 10% and 20%. Joinpoint models were used to summarize annual trend inflections for all reported HFMD incidence, sex-specific incidence, age group counts, and typed serotype counts.

The primary analysis used negative-binomial ITS counterfactual models with month-specific seasonal terms. Following recent counterfactual-prediction guidance, we specified the target observed series, intervention dates, training windows, evaluation metrics, and prediction-interval summaries before interpreting postintervention contrasts [31,32]. For vaccine-era primary models, we used a shorter and more stable preintervention window (January 2013 to May 2016) and specified a linear secular trend to avoid overfitting the short training set. The previous January 2010 to May 2016 quadratic specification was retained as a sensitivity analysis to preserve comparability with previous analyses. For COVID-19–period models, we trained models using data from January 2008 to December 2019 with a linear secular trend. To assess whether the COVID-19 counterfactual depended on including vaccine-era months in the baseline, we also fit a prevaccine sensitivity model using January 2008 to December 2015 as the training window.

Model performance was evaluated on the training period by comparing observed monthly counts with model-fitted counts. We reported mean absolute error (MAE) and root mean square error (RMSE) as primary fit diagnostics because they are expressed in the same count scale as the modeled outcomes; mean absolute percentage error (MAPE) was also tabulated but interpreted cautiously because sparse-typed monthly counts can inflate percentage errors. For each fitted model, we generated postintervention counterfactual predictions and estimated 95% prediction intervals (PIs) by simulating negative-binomial monthly counts from the fitted model and aggregating simulated postintervention counts by year. We summarized observed counts, point predictions, PIs, signed count differences, and relative differences by year. Because province-wide monthly denominators for specimen submission and testing were unavailable, we did not include typing fractions as regressors in the primary models. Instead, we retained previously generated typing-fraction sensitivity analyses as context in Multimedia Appendix 1.

To assess potential severity-related differences within the typed subset, we compared EV71 proportions between severe and mild typed records and fitted adjusted multinomial models of typed serotype category with calendar year, sex, age group, and severity as covariates. To examine the postpandemic age shift, we compared the age composition of reported HFMD cases with known age from 2017 to 2019 vs from 2021 to 2023, performed a chi-square test across age group distributions, and fitted a binomial model comparing the odds that a reported case belonged to the ≥4-year age group of children from 2021 to 2023 vs from 2017 to 2019.

All analyses were performed in R (version 4.5.2; R Foundation for Statistical Computing). Spatiotemporal scan statistics used SaTScan (version 10.2.5; Martin Kulldorff and Information Management Services Inc), and joinpoint analyses used the *nih.joinpoint* R package.

### Ethical Considerations

This study analyzed deidentified routine HFMD surveillance data provided by the Anhui Provincial Center for Disease Control and Prevention as part of the statutory notifiable infectious disease surveillance system. Permission to use the deidentified surveillance data for retrospective analysis was granted by the Anhui Provincial Center for Disease Control and Prevention. The study involved secondary analysis of anonymized records, without direct participant contact, intervention, recruitment, or compensation. No names, identification numbers, addresses, telephone numbers, or other direct personal identifiers were available to the authors, and all public outputs are reported as aggregated temporal or spatial summaries. Under local requirements for routine public health surveillance data [33], separate ethics committee approval and individual informed consent were not required.

## Results

### Overview of Reported and Typed HFMD Cases

From January 2008 through December 2023, Anhui reported 1,292,579 HFMD cases, including 47,990 (3.7%) typed cases. Annual incidence peaked in 2014, with 145,528 reported cases (241 per 100,000 population), while the lowest annual incidence occurred in 2022, with 28,385 reported cases (46 per 100,000 population; Table S1 in Multimedia Appendix 1). Monthly burden retained a bimodal seasonal pattern, with a larger spring–early summer peak and a smaller autumn peak (Figure 1A; Figure S1 in Multimedia Appendix 1). Wavelet analysis identified significant 6- and 12-month periodicities in the reported case series (Figure S2 in Multimedia Appendix 1). The single highest monthly count occurred in July 2023, with 27,489 reported cases.

**Figure 1:**
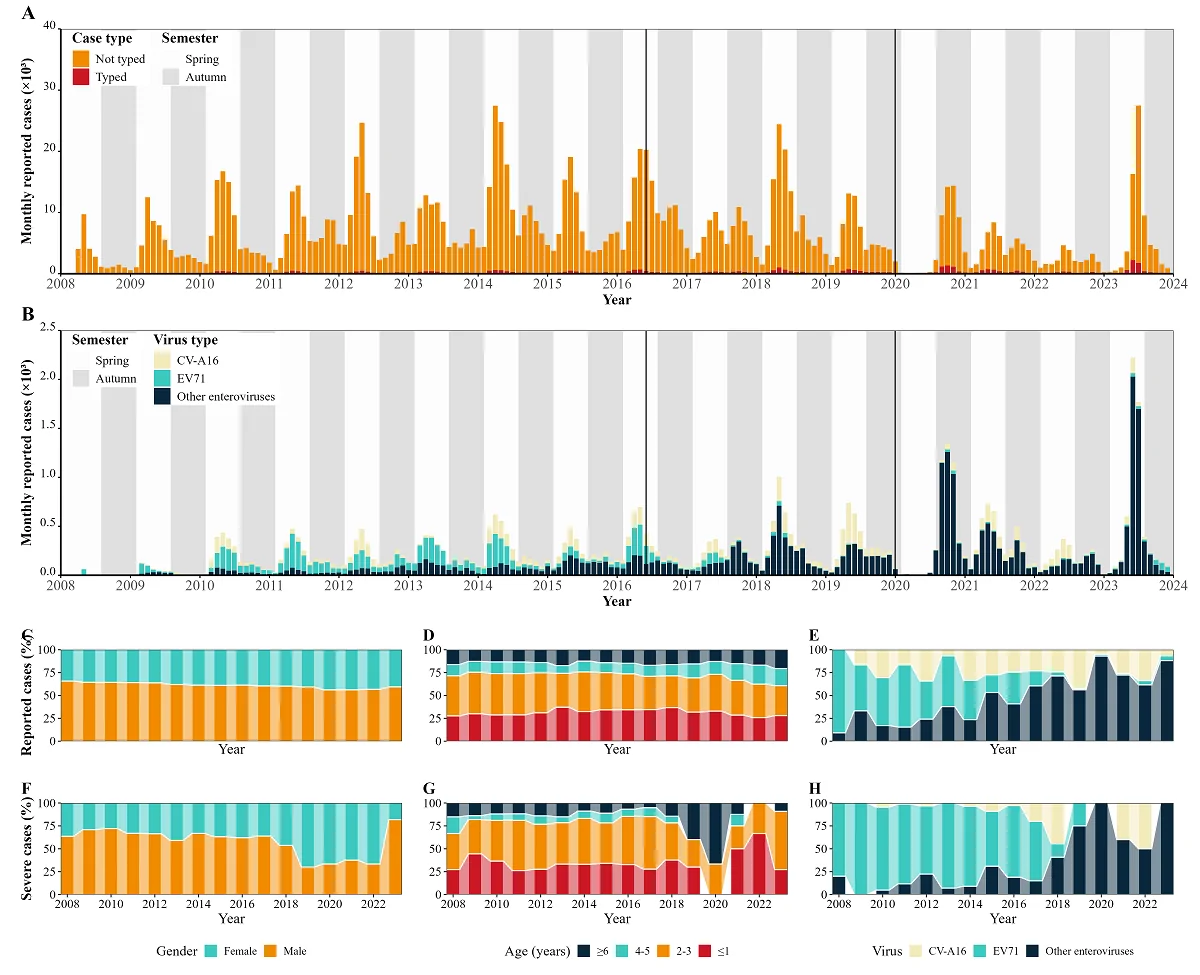
Epidemic curves and case composition of hand, foot, and mouth disease (HFMD) in Anhui Province (2008-2023). (A) Monthly reported HFMD cases, categorized as typed or not typed; (B) monthly typed HFMD cases, displayed as enterovirus 71 (EV71), coxsackievirus A16 (CV-A16), and other enteroviruses; (C, D, and E) annual proportions of cases by sex, age group, and typed serotype, respectively, among all reported cases or typed cases, as appropriate; and (F, G, and H) annual proportions of cases by sex, age group, and typed serotype, respectively, among all severe cases or typed severe cases, as appropriate. Vertical lines indicate June 1, 2016 (EV71 vaccine–era analytic boundary), and January 1, 2020 (start of the COVID-19 period).

HFMD remained concentrated in young children across the full study period. Among the 1,292,579 reported cases, 540,368 (41.8%) occurred among children aged ≤1 year, 517,445 (40%) among children aged 2 to 3 years, and 234,766 (18.2%) among children aged ≥4 years. Male participants accounted for 791,458 (61.2%) reported cases, compared with 501,121 (38.8%) among female participants. Overall, 3064/1,292,579 (0.24%) severe cases were recorded during 2008 to 2023. Severe cases became rare after 2018, with 10/70,759 (0.014%) severe cases in 2019 and 3 to 11 severe cases annually from 2020 to 2023. The death-date field identified 123 deaths during 2008 to 2023, corresponding to 9.52 deaths per 100,000 reported cases; no deaths were recorded during 2020 to 2023 (Figure S5 and Tables S6 and S7 in Multimedia Appendix 1).

### Typed Serotype Composition

Among typed cases, the January 2008 to May 2016 period included 18,534 typed detections: EV71 represented 8479 (45.8%), CV-A16 represented 4568 (24.6%), and other enteroviruses represented 5487 (29.6%; Figure 1B; Table S5 in Multimedia Appendix 1). The immediate postrollout prepandemic period (June 2016 to December 2019) included 12,526 typed detections: EV71 accounted for 1114 (8.9%), CV-A16 accounted for 3718 (29.7%), and other enteroviruses accounted for 7694 (61.4%). The COVID-19 period (January 2020 to December 2023) included 16,930 typed detections: other enteroviruses accounted for 13,848 (81.8%), EV71 accounted for 578 (3.4%), and CV-A16 accounted for 2504 (14.8%) (Figure S3 in Multimedia Appendix 1).

The annual proportion of reported cases with typing increased from 0.3% (76/26,359) of reported cases in 2008 to 5.5% (3919/70,759) in 2019, 8.4% (4429/52,712) in 2020, and 8.4% (5884/70,288) in 2023. In annual typed composition, EV71 accounted for 90.8% (69/76) of typed records in 2008, 34.6% (1266/3664) in 2016, 16.3% (470/2885) in 2017, and 1.6% (63/3919) in 2019. Other enteroviruses accounted for 93.1% (4122/4429) of typed records in 2020 and 88.1% (5186/5884) in 2023 (Table S5 in Multimedia Appendix 1).

In an adjusted multinomial model of typed records, severe cases were more likely than mild cases to be typed as EV71 rather than other enteroviruses (relative risk ratio 7.08, 95% CI 6.01-8.34; Table S5, and Table S8 in Multimedia Appendix 1). Older age groups (2-3 years and ≥4 years) were also more likely than the reference group (≤1 year) to be typed as EV71 or CV-A16 rather than other enteroviruses, while male sex had little association with EV71 classification (relative risk ratio 1.01, 95% CI 0.95-1.07).

### Secondary Spatial and Temporal Analyses

Annual incidence maps showed persistent geographic heterogeneity across Anhui (Figure 2; Figure S4 in Multimedia Appendix 1). Retrospective spatiotemporal scan statistics identified 26 cluster windows, of which 25 were significant at *P*<.05 (Table S2 in Multimedia Appendix 1). Wuhu was the most frequently identified cluster center (11 windows), followed by Huangshan, Ma’anshan, and Bengbu (4 windows each). The largest log-likelihood cluster was centered in Huangshan from April to October 2014, with 18,324 observed cases vs 5195 expected cases (relative risk 3.56). The highest relative risk cluster was centered in Ma’anshan from April to June 2012, with 11,585 observed cases vs 2444 expected cases (relative risk 4.77). Annual global Moran *I* was significant in 2008 (Moran *I*=0.43; *P*<.001), 2016 (Moran *I*=0.38; *P*=.002), and 2021 (Moran *I*=0.26; *P*=.02), but not in most other years (Table S3 in Multimedia Appendix 1).

**Figure 2:**
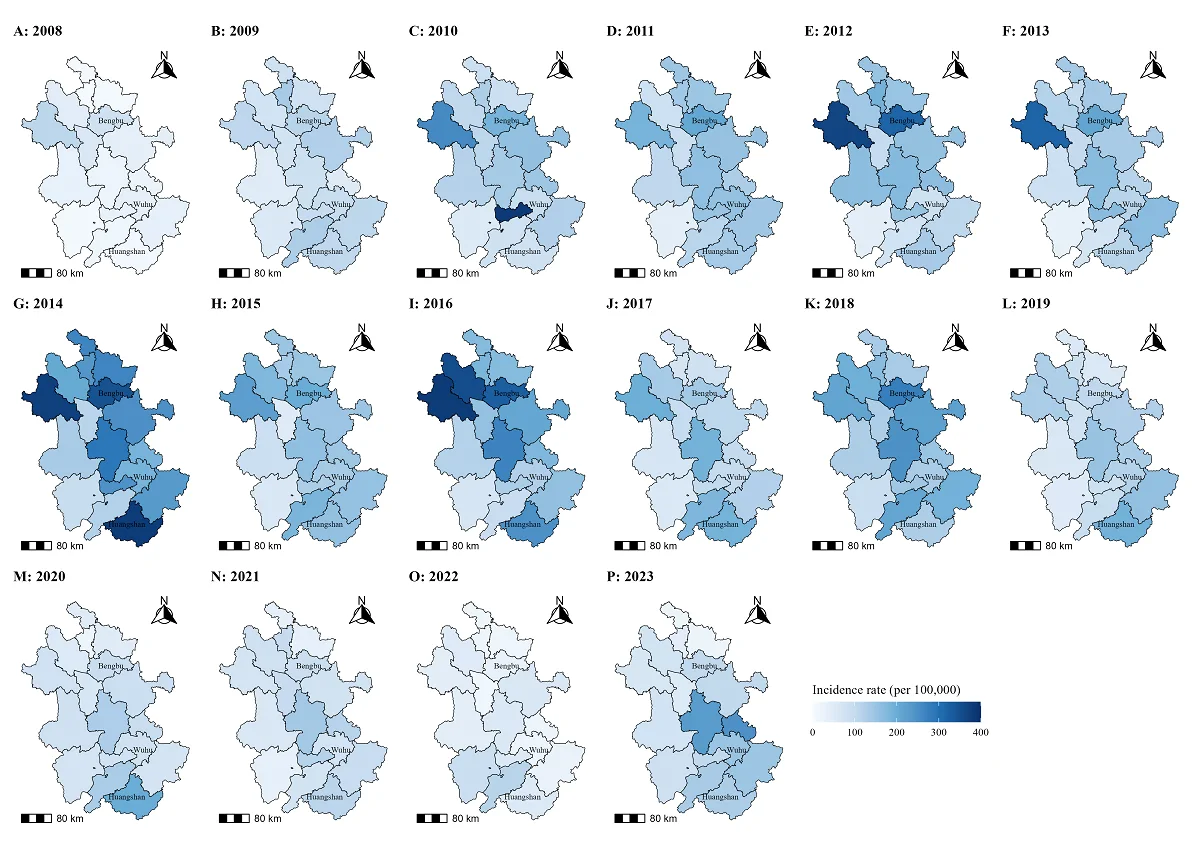
Annual incidence maps of reported hand, foot, and mouth disease (HFMD) in Anhui Province, China (2008-2023). Each panel shows the incidence rate for each prefecture-level city in the corresponding year. Labels for Bengbu, Wuhu, and Huangshan are added as spatial references because these locations are discussed in the text.

County-level typed-dominance summaries showed that apparent EV71-dominant county-years decreased over time, while other enterovirus–dominant county-years became more frequent (Figure 3; Figure S8 in Multimedia Appendix 1). County-year observations with <10 typed cases were masked. Dominant-serotype labels indicate only the largest typed category in a county-year and do not measure the margin between the leading and second-leading typed categories.

**Figure 3:**
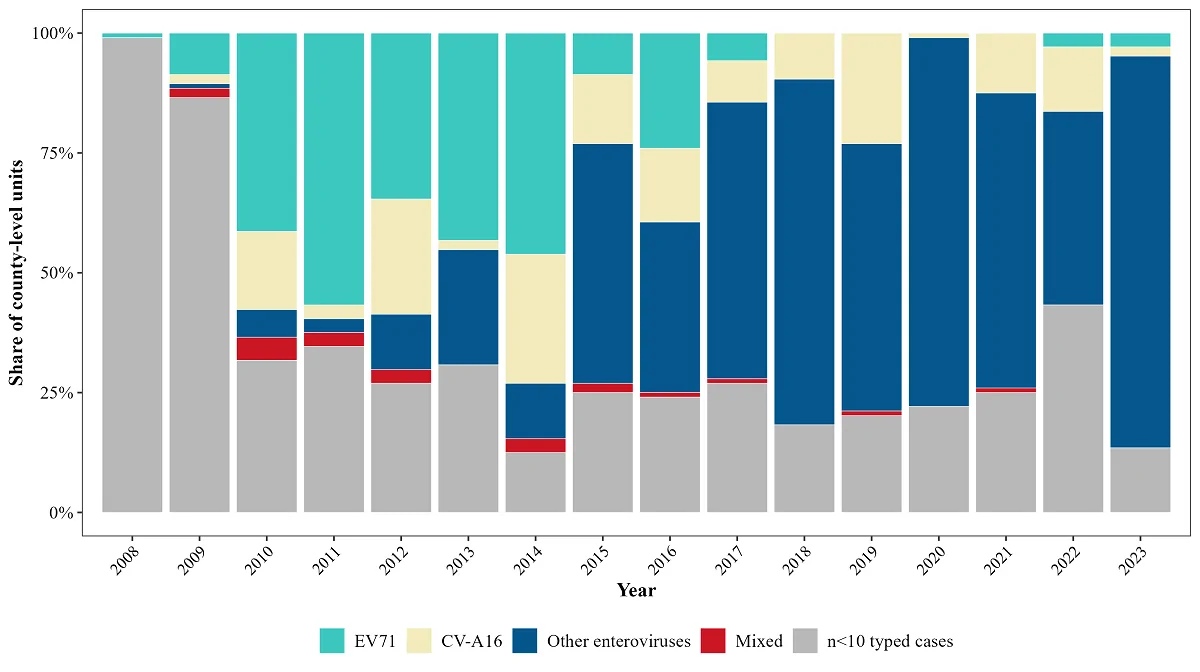
Annual county-level dominance of typed serotype after masking sparse observations in Anhui Province, China (2008-2023). The 100% stacked bar chart shows the share of county-level units classified as enterovirus 71 (EV71)–dominant, coxsackievirus A16 (CV-A16)–dominant, other enterovirus–dominant, mixed, or masked because of <10 typed cases. Counties with <10 typed cases in a given year were not assigned a dominant typed serotype and are shown as masked observations. Dominance indicates the largest typed category in a county-year and should not be interpreted as evidence that one serotype was substantially more frequent than the next most common typed serotype.

Joinpoint regression suggested a province-wide inflection around 2014 for all reported HFMD incidence and a sustained decline thereafter, whereas typed EV71 counts showed a downward long-term pattern after the early years of laboratory expansion (Figure 4). Count-regression sensitivity analyses for annual typed counts showed a negative annual trend for EV71 in the negative-binomial model (annual percentage change −14.4%, 95% CI −22% to −6%), a positive annual trend for other enteroviruses (annual percentage change 19.5%, 95% CI 19.2%-19.9%), and no statistically clear annual trend for CV-A16.

**Figure 4:**
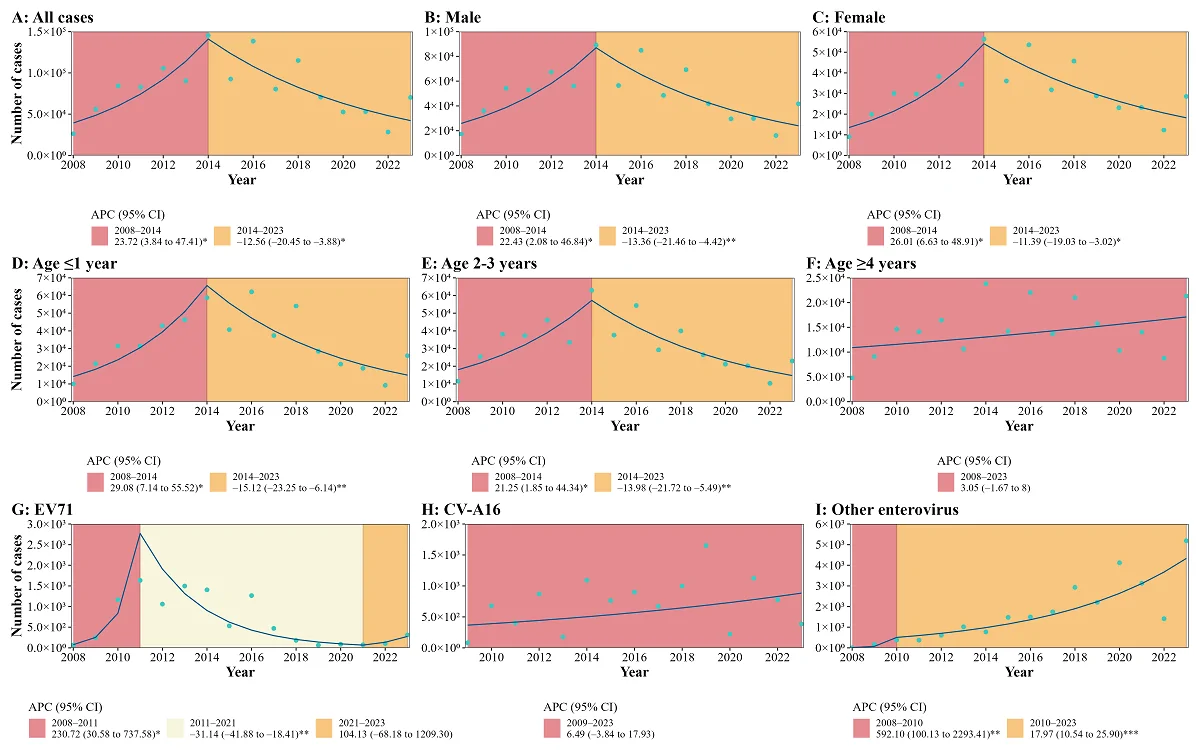
Joinpoint regression of annual hand, foot, and mouth disease (HFMD) trends in Anhui Province, China (2008-2023). (A) Overall incidence, (B) male incidence, (C) female incidence, (D) age ≤1 year, (E) age 2 to 3 years, (F) age ≥4 years, (G) typed enterovirus 71 (EV71) counts, (H) typed coxsackievirus A16 (CV-A16) counts, and (I) typed other enterovirus counts. Dots indicate observed values, and lines indicate fitted segments with 95% CIs. APC: annual percent change. P<0.05, P<0.01, P<0.001.

### ITS Analyses of Vaccine-Era Models

Primary vaccine-era ITS models were trained using data from January 2013 to May 2016 and included a linear secular trend (Figures 5A and 5B). Training-period MAE and RMSE were 2065.5 and 2950.6 monthly cases, respectively, for all reported HFMD cases and 35.5 and 53.4 monthly cases, respectively, for typed EV71 cases. Observed annual typed EV71 counts remained below the predicted trajectory throughout 2017 to 2019 (Figures 5C and 5D; PI summaries are provided in Table S4 in Multimedia Appendix 1): 470 observed vs 659 predicted cases (95% PI 467–933) in 2017, 181 vs 531 (95% PI 379–798) in 2018, and 63 vs 427 (95% PI 305–662) in 2019. These corresponded to annual relative differences of −28.7%, −65.9%, and −85.2%, respectively.

For all reported HFMD cases, observed burden was lower than predicted in 2017 (80,344 observed vs 104,712 predicted; 95% PI 84,388-129,080) and 2019 (70,759 observed vs 101,494 predicted; 95% PI 82,487-132,654), but higher than the point prediction in 2018 (115,029 observed vs 103,091 predicted; 95% PI 82,975-130,561; Figures 5A and 5B). Secondary typed series showed lower-than-predicted CV-A16 counts in 2017 to 2019, while typed other enterovirus counts were below prediction in 2017 and 2019 but above the point prediction in 2018 (Table S3, Table S4 and Figure S7 in Multimedia Appendix 1).

Sensitivity analyses using the previous January 2010 to May 2016 quadratic training window preserved the same direction of post-2016 negative divergence for typed EV71, although effect magnitudes were attenuated compared with the primary model (Table S9 in Multimedia Appendix 1). The longer-window specification yielded 2017 to 2019 typed EV71 counts that were 17%, 49.7%, and 70% below prediction, respectively, compared with 28.7%, 65.9%, and 85.2% in the primary short-window model.

**Figure 5:**
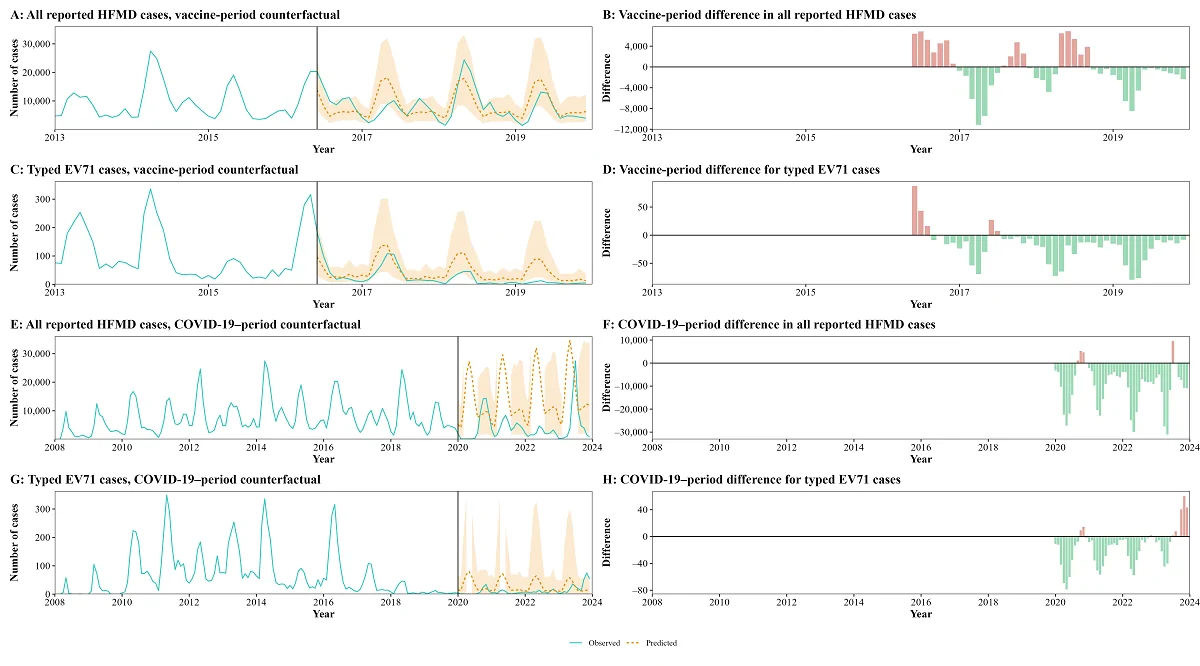
Primary ecological interrupted time series counterfactual analyses for hand, foot, and mouth disease (HFMD) surveillance in Anhui Province, China. The primary models focused on all reported HFMD cases and typed enterovirus 71 (EV71) cases. Panels show observed monthly case counts, predicted counterfactual trajectories, and 95% prediction intervals (PIs), along with postintervention difference plots. (A-D) Vaccine-era models (trained January 2013–May 2016) showing (A, B) all reported HFMD cases and (C, D) typed EV71 cases. (E-H) COVID-19–period models (trained January 2008–December 2019) showing (E, F) all reported HFMD cases and (G, H) typed EV71 cases.

### ITS Analyses of COVID-19–Period Models

COVID-19–period ITS models were trained from January 2008 to December 2019. Training-period MAE and RMSE were 2756.2 and 3821.3 monthly cases, respectively, for all reported HFMD and 47.4 and 70.0 monthly cases, respectively, for typed EV71. All reported HFMD cases remained below the pre-2020 counterfactual throughout 2020 to 2023 (Figures 5E and 5F; Table S4 in Multimedia Appendix 1): 52,712 observed vs 149,675 predicted (95% PI 96,603-222,919) in 2020, 53,122 vs 162,121 (95% PI 105,863-243,423) in 2021, 28,385 vs 175,601 (95% PI 111,465-268,202) in 2022, and 70,288 vs 190,201 (95% PI 127,746-284,820) in 2023. The corresponding annual relative differences were −64.8%, −67.2%, −83.8%, and −63%.

Typed EV71 counts were also below predicted values in 2020 to 2022 (Figure 5G and 5H): 87 observed vs 395 predicted in 2020, 72 vs 355 in 2021, and 106 vs 319 in 2022. In 2023, typed EV71 was slightly above the point prediction but remained within the 95% PI (313 observed vs 287 predicted; 95% PI 113-678). Typed CV-A16 and typed other enterovirus counts were below predicted values throughout 2020 to 2023 in the primary COVID-19–period models (Table S4 and Figure S7 in Multimedia Appendix 1).

A sensitivity model trained only on the prevaccine period (January 2008 to December 2015) yielded the same direction of post-2020 suppression for all reported HFMD and the typed serotype series (Table S10 in Multimedia Appendix 1).

### Postpandemic Age Shift

The age composition of reported HFMD cases shifted upward after the most restrictive pandemic period (Figure S6 in Multimedia Appendix 1). Among cases with known age, the proportion of children aged ≥4 years increased from 19% (50,503/266,132) in 2017 to 2019 to 29.1% (44,133/151,795) in 2021 to 2023, while the proportion aged ≤1 year decreased from 45% (119,868/266,132) to 35.6% (54,102/151,795). The ≥4-year age group accounted for 26.5% (14,060/53,122) in 2021, 30.9% (8765/28,385) in 2022, and 30.3% (21,308/70,288) in 2023.

The age distribution comparison between 2017 and 2019 and 2021 and 2023 was statistically significant (χ^2^_2_=6417.0; *P*<.001). The odds that a reported case belonged to the ≥4-year age group were higher in 2021 to 2023 than in 2017 to 2019 (odds ratio 1.75, 95% CI 1.72-1.78).

## Discussion

### Principal Results

This study should be read as a long-term surveillance analysis, not as a formal assessment of vaccination-related protection. The main findings were that typed EV71 surveillance signals declined after EV71 vaccine introduction, overall reported HFMD burden did not show a uniform postrollout decrease, and the COVID-19 pandemic created a large disruption in the same ecological time series. The revised analyses also showed that severe and fatal outcomes became rare in the later years, that other enterovirus records became dominant within the typed subset, that spatial clustering persisted in selected years and locations, and that the postpandemic rebound was accompanied by an older age distribution among reported cases.

The most important interpretive boundary is the typed subset. Only 3.7% (47,990/1,292,579) reported cases had etiologic typing. Individual vaccination records, province-wide vaccine coverage histories, testing policy metadata, and laboratory platform-change records were unavailable. In addition, severe cases were more likely than mild cases to be classified as EV71 rather than other enteroviruses among typed records. These features mean that the typed EV71 series cannot be treated as an unbiased proxy for community serotype incidence.

### Interpretation of Typed EV71 and Vaccine-Era Signals

Within those limits, the vaccine-era primary ITS models showed a sustained negative divergence of typed EV71 counts below the counterfactual trajectory during 2017 to 2019. This pattern is directionally consistent with reports from other Chinese provinces after EV71 vaccine introduction [6,24-26]. However, the magnitude of our typed EV71 counterfactual contrast was larger than several published population-level estimates. Guangdong reported a 41.4% reduction in EV71-associated HFMD in 2017 to 2019 with increasing vaccine coverage, and Jiangsu reported an estimated 45.6% reduction with a wide credible interval [24,25]. By comparison, our primary short-window model estimated that typed EV71 counts were 28.7%, 65.9%, and 85.2% below predicted values in 2017, 2018, and 2019, respectively, while the longer-window sensitivity analysis estimated smaller reductions of 17%, 49.7%, and 70%, respectively.

Several mechanisms could make the Anhui-typed EV71 contrast appear larger than a true population-level vaccine effect. Early typing fractions were low, the typed subset may have overrepresented severe cases, severe EV71-associated disease may have been preferentially depleted after vaccine rollout, and changes in specimen submission or laboratory platforms could have altered the case mix entering the typed series. For that reason, the relevant message is not that Anhui had unusually high vaccine impact, but that routine typed surveillance recorded a sustained decline in EV71 signals while the overall reported HFMD burden remained substantial.

The aggregated other enteroviruses category requires similar caution. In this dataset, other enteroviruses were recorded only as typed results that were neither EV71 nor CV-A16. That grouping does not identify which serotypes drove the increase, and it could be sensitive to changes in diagnostic breadth over time [12,15,16]. If multiplex polymerase chain reaction became more common during the later study period, previously underdetected CV-A6, CV-A10, or other enteroviruses could have been more readily classified into the other enteroviruses group [12-16]. We therefore interpret the rise of other enteroviruses as a change in typed surveillance composition rather than definitive evidence of population-level serotype replacement.

### COVID-19 Disruption and Postpandemic Age Shift

The COVID-19 period created a distinct ecological disruption. All reported HFMD counts remained far below the pre-2020 counterfactual through 2023, consistent with documented suppression of contact-based infectious diseases during intensive NPIs [17-22]. The same direction held when the COVID-19 counterfactual was anchored only to prevaccine data, reducing concern that the pandemic signal was an artifact of including vaccine-era months in the baseline. At the same time, the postpandemic age composition shifted toward older children. This local age shift is compatible with an immunity-gap or immunity-debt interpretation, but the evidence remains indirect because the surveillance system does not measure susceptibility or prior infection history [23,34-37]. We therefore present this mechanism as a plausible interpretation supported by age-structured surveillance patterns, not as a proven causal explanation. A pre-vaccine meta-analysis from China documented increasing EV71 antibody seroprevalence with age among preschool children [38], providing historical context for age-related immunity rather than direct evidence of a pandemic-related immunity gap. From a policy perspective, the age shift suggests that postpandemic HFMD preparedness should not focus only on infants and very young preschool children. If linked vaccination and coverage data become available, Anhui could evaluate whether unvaccinated older preschool or school-entry children would benefit from catch-up EV71 vaccination; however, such a strategy cannot be recommended from the current ecological data alone, especially because absolute typed EV71 counts remained low and the vaccine does not protect against non-EV71 HFMD [3-8].

### Spatial and Seasonal Surveillance Findings

The secondary spatial and seasonal analyses add descriptive context to the ITS findings. The bimodal seasonal pattern and significant 6- and 12-month periodicities indicate that seasonal forcing remained a recurring feature of Anhui HFMD activity across the long surveillance window [34,35]. Spatiotemporal scan statistics repeatedly identified clusters centered in Wuhu, Huangshan, Bengbu, and Ma’anshan, but annual Moran *I* was significant only in selected years. These findings indicate spatial heterogeneity in reported burden, but they do not identify causal drivers such as mobility, school calendars, climate, health care access, socioeconomic conditions, or vaccine uptake [34,35].

County-level typed-dominance maps should be read even more cautiously than incidence maps. The dominance label is categorical, depends only on typed records, and can be unstable when typed counts are small. Masking county-years with <10 typed cases reduces the most extreme instability, but it does not remove selection into testing or measure how close the leading typed category was to the next most common category. These maps are therefore best used to flag areas for enhanced surveillance rather than to infer local replacement of serotypes.

### Comparison With Prior Work

Our findings overlap with prior Chinese surveillance studies showing reduced EV71 detection after vaccine introduction and persistent circulation of non-EV71 enteroviruses [6,9,19,24-26]. The added value of the current study is not mechanistic discovery or broad causal generalization beyond Anhui. Instead, it is the explicit framing of what surveillance data can and cannot support when typing is sparse, when the non-EV71 category is aggregated, and when the COVID-19 period interrupts the same time series [10,12,13,27]. This framing aligns more closely with public health surveillance priorities than with causal inference regarding vaccine impact.

The findings also align with broader reports that COVID-19 pandemic control measures suppressed multiple pediatric and contact-transmitted infections, followed in some settings by rebound or altered age distributions when contact patterns normalized [17-23]. Anhui adds a province-level example in which pandemic-era suppression occurred against the background of an already changing EV71 vaccine–era surveillance series.

### Limitations

This study has several limitations. First, the absence of individual vaccination records and province-wide vaccine coverage histories means that we could not account for actual vaccine uptake, estimate vaccine effectiveness, or distinguish vaccine-program effects from other contemporaneous changes [10,24,25]. Second, the surveillance system was passive, so reporting may have been influenced by health care–seeking behavior and pandemic-era service disruption. Third, etiologic typing remained sparse and likely nonrandom, with severe cases more likely to be typed and the association between severity and typing changing over time. We did not have province-wide metadata on specimen submission criteria, the balance between mild and severe cases selected for testing, or annual changes in laboratory platform use [10-13]. Fourth, the aggregated other enteroviruses category may have been influenced by diagnostic drift; wider adoption of multiplex polymerase chain reaction could make CV-A6, CV-A10, or other previously underdetected serotypes more visible without proving population-level niche replacement [12-16,27]. Fifth, because the postvaccine period and the COVID-19 period are contiguous in the same ecological series, no stable interdisruption interval was available for formal statistical separation of their independent effects. Sixth, although the death-date field allowed us to summarize fatal outcomes, the surveillance extract did not include follow-up information on neurological sequelae, so we could not estimate the proportion of survivors with neurological sequelae after severe HFMD. Finally, county-level dominant-serotype maps are sensitive to sparse-typed counts even after masking county-year cells with <10 typed cases.

### Public Health Implications

The practical contribution of this analysis is a surveillance blueprint rather than an estimate of vaccine effectiveness. Future HFMD surveillance would be more interpretable if specimen submission rules were standardized across sites and years; if sentinel hospitals routinely used multiplex polymerase chain reaction or sequencing capable of distinguishing CV-A6, CV-A10, and other non-EV71 serotypes; and if surveillance extracts preserved the denominator of submitted and successfully tested specimens [11-16]. Linkage to immunization registries would be needed before the same surveillance system could support vaccine effectiveness analyses [10,24,25]. Wastewater or environmental surveillance could provide an additional low-cost early-warning stream for non-EV71 enteroviruses, especially when clinical typing remains sparse [39,40].

### Conclusions

Routine HFMD surveillance in Anhui showed a decline in typed EV71 surveillance signals after EV71 vaccine introduction, a concurrent rise in typed other enterovirus detections, and strong pandemic-era suppression of overall HFMD burden followed by partial rebound and an older-age shift. These findings support extending surveillance priorities beyond EV71 alone. For practice, this means standardizing specimen submission rules, introducing routine multiplex polymerase chain reaction in eligible sentinel hospitals, preserving testing denominators and vaccination histories in surveillance extracts, and considering wastewater surveillance as a lower-cost supplementary early-warning approach. For interpretation, this requires maintaining ecological humility: changes in typed surveillance composition can illuminate temporal patterns but cannot, on their own, establish vaccine-attributable protection or population-level serotype replacement.

## Appendix

Multimedia Appendix 1 Supplementary figures and tables for descriptive epidemiology, spatial and temporal analyses, and interrupted time series analyses of hand, foot, and mouth disease in Anhui, China.

## Abbreviations

CV-A6: coxsackievirus A6
CV-A10: coxsackievirus A10
CV-A16: coxsackievirus A16
EV71: enterovirus 71
HFMD: hand, foot, and mouth disease
ITS: interrupted time series
MAE: mean absolute error
MAPE: mean absolute percentage error
NPI: nonpharmaceutical intervention
PI: prediction interval
RMSE: root mean square error
STROBE: Strengthening the Reporting of Observational Studies in Epidemiology

## Data Availability

Supplementary figures and tables are provided in Multimedia Appendix 1. Aggregated analytic data, analysis scripts, and analytic outputs supporting this study are publicly available in our GitHub repository [41]. The individual-level surveillance records are not publicly available because they contain sensitive statutory notifiable disease surveillance information and are governed by the Anhui Provincial Center for Disease Control and Prevention; requests for additional access would require approval from the data custodian. Publicly released materials contain aggregated temporal and spatial counts rather than individual-level surveillance records.
