# Supplementary material for: Hand, Foot, and Mouth Disease Surveillance in Anhui, After Introduction of the Enterovirus 71 Vaccine and During the COVID-19 Pandemic: Ecological Interrupted Time Series Study
Source: JMIR Public Health Surveill. 2026 Sep 24;12:e99663. doi: 10.2196/99663 (PMC13650459; doi:10.2196/99663)
Supplement: Supplementary file 1 [file publichealth_v12i1e99663_app1.docx]

Supplementary Appendix

Hand, Foot, and Mouth Disease Surveillance in Anhui, China, After Introduction of the Enterovirus 71 Vaccine and During the COVID-19 Pandemic: Ecological Interrupted Time Series Study

Kangguo Li et al.

**Figure S1. Seasonal patterns of HFMD incidence across prefecture-level cities in Anhui Province, 2008-2023.**

**Figure S2. Global wavelet power spectrum of reported HFMD cases in Anhui Province.**

**Figure S3. Period-stratified incidence maps of reported HFMD in Anhui Province.**

**Figure S4. Yearly spatiotemporal scan relative-risk maps for HFMD clusters in Anhui Province.**

**Figure S5. Binomial ITS counterfactual analysis of severe HFMD proportion.**

**Figure S6. Annual age composition of reported HFMD cases with known age.**

**Figure S7. Counterfactual trajectories for typed CV-A16 and typed other-enterovirus series in the vaccine-era and COVID-19-period models.**

**Figure S8. County-level typed-serotype dominance maps after masking sparse observations, Anhui Province, 2008-2023. Colors match Figure 3; gray indicates county-year observations with typed n<10. Dominance indicates the largest typed category within a county-year and should not be interpreted as a large margin between categories.**

**Table S1. Annual epidemiologic and demographic characteristics of HFMD in Anhui Province, 2010-2023**

| Characteristic | 2010 | 2011 | 2012 | 2013 | 2014 | 2015 | 2016 | 2017 | 2018 | 2019 | 2020 | 2021 | 2022 | 2023 |
| --- | --- | --- | --- | --- | --- | --- | --- | --- | --- | --- | --- | --- | --- | --- |
| Total | 84323(100%) | 82739(100%) | 105554(100%) | 90447(100%) | 145528(100%) | 92572(100%) | 138493(100%) | 80344(100%) | 115029(100%) | 70759(100%) | 52712(100%) | 53122(100%) | 28385(100%) | 70288(100%) |
| Age |  |  |  |  |  |  |  |  |  |  |  |  |  |  |
| <12 months | 7315(8.68%) | 7537(9.11%) | 10197(9.66%) | 12715(14.06%) | 11577(7.96%) | 9005(9.73%) | 14510(10.48%) | 9649(12.01%) | 11780(10.24%) | 5985(8.46%) | 3913(7.42%) | 3693(6.95%) | 1924(6.78%) | 6331(9.01%) |
| 12-23 months | 24205(28.71%) | 23776(28.75%) | 32787(31.07%) | 33599(37.16%) | 47197(32.43%) | 31722(34.27%) | 47589(34.37%) | 27721(34.50%) | 42233(36.71%) | 22500(31.81%) | 17312(32.85%) | 15151(28.52%) | 7325(25.81%) | 19678(28.01%) |
| 24-35 months | 23684(28.09%) | 23012(27.81%) | 28990(27.47%) | 21643(23.93%) | 39653(27.24%) | 22946(24.79%) | 32630(23.56%) | 16823(20.94%) | 23313(20.27%) | 14390(20.33%) | 11065(20.99%) | 10151(19.11%) | 4830(17.02%) | 12196(17.35%) |
| 36-59 months | 21786(25.83%) | 21427(25.89%) | 25239(23.90%) | 17154(18.96%) | 34666(23.83%) | 21376(23.09%) | 32701(23.61%) | 18911(23.54%) | 25875(22.49%) | 19127(27.02%) | 15344(29.11%) | 16412(30.91%) | 8592(30.28%) | 18957(26.96%) |
| 5-9 years | 6448(7.65%) | 6207(7.50%) | 7471(7.08%) | 4559(5.04%) | 10966(7.54%) | 6568(7.10%) | 9750(7.04%) | 6160(7.67%) | 10085(8.77%) | 7739(10.94%) | 4059(7.70%) | 6592(12.41%) | 4334(15.26%) | 10783(15.34%) |
| >9 years | 885(1.05%) | 780(0.94%) | 870(0.82%) | 777(0.86%) | 1469(1.01%) | 955(1.03%) | 1313(0.95%) | 1080(1.34%) | 1743(1.52%) | 1018(1.44%) | 1019(1.93%) | 1123(2.11%) | 1380(4.86%) | 2343(3.33%) |
| Gender |  |  |  |  |  |  |  |  |  |  |  |  |  |  |
| male | 54257(64.34%) | 52957(64.01%) | 67333(63.80%) | 56062(61.98%) | 89154(61.26%) | 56487(61.02%) | 84863(61.28%) | 48537(60.41%) | 69303(60.24%) | 41918(59.23%) | 29578(56.13%) | 29838(56.19%) | 16109(56.74%) | 41709(59.35%) |
| female | 30066(35.66%) | 29782(35.99%) | 38211(36.20%) | 34385(38.02%) | 56374(38.74%) | 36085(38.98%) | 53630(38.72%) | 31807(39.59%) | 45726(39.76%) | 28841(40.77%) | 23134(43.87%) | 23284(43.81%) | 12276(43.26%) | 28579(40.65%) |
| Severity |  |  |  |  |  |  |  |  |  |  |  |  |  |  |
| mild | 83739(99.31%) | 82307(99.48%) | 105304(99.76%) | 90215(99.74%) | 145077(99.69%) | 92393(99.81%) | 137921(99.59%) | 80189(99.81%) | 114960(99.94%) | 70749(99.99%) | 52709(99.99%) | 53114(99.98%) | 28382(99.99%) | 70277(99.98%) |
| severe | 584(0.69%) | 432(0.52%) | 250(0.24%) | 232(0.26%) | 451(0.31%) | 179(0.19%) | 572(0.41%) | 155(0.19%) | 69(0.06%) | 10(0.01%) | 3(0.01%) | 8(0.02%) | 3(0.01%) | 11(0.02%) |
| Virus |  |  |  |  |  |  |  |  |  |  |  |  |  |  |
| Total | 2229(100%) | 2403(100%) | 2542(100%) | 2692(100%) | 3268(100%) | 2770(100%) | 3664(100%) | 2885(100%) | 4112(100%) | 3919(100%) | 4429(100%) | 4323(100%) | 2294(100%) | 5884(100%) |
| EV71 | 1166(52.31%) | 1634(68.00%) | 1059(41.66%) | 1497(55.61%) | 1405(42.99%) | 532(19.21%) | 1266(34.55%) | 470(16.29%) | 181(4.40%) | 63(1.61%) | 87(1.96%) | 72(1.67%) | 106(4.62%) | 313(5.32%) |
| CV-A16 | 681(30.55%) | 398(16.56%) | 870(34.23%) | 177(6.58%) | 1092(33.41%) | 763(27.55%) | 900(24.56%) | 670(23.22%) | 999(24.29%) | 1653(42.18%) | 220(4.97%) | 1123(25.98%) | 776(33.83%) | 385(6.54%) |
| Others | 382(17.14%) | 371(15.44%) | 613(24.11%) | 1018(37.82%) | 771(23.59%) | 1475(53.25%) | 1498(40.88%) | 1745(60.49%) | 2932(71.30%) | 2203(56.21%) | 4122(93.07%) | 3128(72.36%) | 1412(61.55%) | 5186(88.14%) |

Note: Due to rounding, the sum of percentages may not exactly equal 100%. To ensure the total is 100%, the rounding discrepancy (within 0.1 percentage points) was adjusted to the category with the largest proportion. This adjustment does not affect interpretation. Table S1 begins in 2010 for presentation consistency because etiological records in 2008-2009 were sparse and unstable for annual virus-composition tabulation. The overall study period analyzed in the manuscript remained 2008-2023.

**Table S2. Spatiotemporal cluster detection summary**

| Name | Radius | Start date | End date | P value | Observed | Expected | Relative risk | Year | LLR |
| --- | --- | --- | --- | --- | --- | --- | --- | --- | --- |
| Huangshan | 85.99 | 2009/4/1 | 2009/6/30 | <.001 | 4585 | 2173.35 | 2.11 | 2009 | 1013.41 |
| Ma’anshan | 108.75 | 2010/4/1 | 2010/6/30 | <.001 | 6864 | 2529.56 | 2.72 | 2010 | 2524.85 |
| Wuhu | 108.11 | 2010/4/1 | 2010/6/30 | <.001 | 2614 | 1658.98 | 1.58 | 2010 | 233.87 |
| Bengbu | 85.99 | 2011/5/1 | 2011/7/31 | <.001 | 7531 | 2908.28 | 2.60 | 2011 | 2551.11 |
| Wuhu | 108.11 | 2011/5/1 | 2011/7/31 | <.001 | 3656 | 1640.59 | 2.23 | 2011 | 915.78 |
| Ma’anshan | 108.75 | 2012/4/1 | 2012/6/30 | <.001 | 11585 | 2444.12 | 4.77 | 2012 | 8918.33 |
| Wuhu | 108.11 | 2012/5/1 | 2012/6/30 | <.001 | 2161 | 1091.44 | 1.98 | 2012 | 407.00 |
| Wuhu | 108.11 | 2013/4/1 | 2013/6/30 | <.001 | 3029 | 1635.02 | 1.85 | 2013 | 474.39 |
| Huangshan | 85.99 | 2014/4/1 | 2014/10/31 | <.001 | 18324 | 5195.25 | 3.56 | 2014 | 10035.66 |
| Wuhu | 108.11 | 2014/9/1 | 2014/11/30 | <.001 | 2674 | 1644.51 | 1.63 | 2014 | 270.84 |
| Bengbu | 85.99 | 2015/4/1 | 2015/6/30 | <.001 | 8393 | 2967.13 | 2.84 | 2015 | 3312.70 |
| Wuhu | 108.11 | 2015/4/1 | 2015/6/30 | <.001 | 4918 | 1658.78 | 2.97 | 2015 | 2089.89 |
| Huangshan | 85.99 | 2016/4/1 | 2016/11/30 | <.001 | 15812 | 6044.12 | 2.64 | 2016 | 5475.67 |
| Wuhu | 108.11 | 2016/5/1 | 2016/6/30 | <.001 | 1805 | 1116.78 | 1.62 | 2016 | 178.56 |
| Ma’anshan | 70.61 | 2017/10/1 | 2017/11/30 | .55 | 1378 | 1240.25 | 1.11 | 2017 | 7.39 |
| Huaibei | 42.22 | 2018/4/1 | 2018/6/30 | <.001 | 8425 | 1839.72 | 4.60 | 2018 | 6251.04 |
| Wuhu | 108.11 | 2018/5/1 | 2018/7/31 | <.001 | 5128 | 1720.46 | 2.99 | 2018 | 2197.39 |
| Wuhu | 108.11 | 2019/5/1 | 2019/6/30 | <.001 | 3313 | 1149.54 | 2.89 | 2019 | 1345.14 |
| Bengbu | 85.99 | 2019/5/1 | 2019/7/31 | <.001 | 5479 | 3117.63 | 1.76 | 2019 | 730.14 |
| Wuhu | 108.11 | 2020/10/1 | 2020/12/31 | <.001 | 4030 | 1644.26 | 2.46 | 2020 | 1229.28 |
| Ma’anshan | 70.61 | 2020/10/1 | 2020/12/31 | <.001 | 4206 | 1798.92 | 2.34 | 2020 | 1167.45 |
| Chuzhou | 45.66 | 2020/10/1 | 2020/11/30 | <.001 | 4140 | 1781.28 | 2.33 | 2020 | 1134.97 |
| Bengbu | 85.99 | 2021/5/1 | 2021/6/30 | <.001 | 2391 | 1917.73 | 1.25 | 2021 | 54.20 |
| Wuhu | 108.11 | 2023/6/1 | 2023/7/31 | <.001 | 4847 | 1109.36 | 4.38 | 2023 | 3415.10 |
| Chuzhou | 45.66 | 2023/7/1 | 2023/8/31 | <.001 | 5051 | 1814.19 | 2.79 | 2023 | 1939.24 |
| Huangshan | 85.99 | 2023/7/1 | 2023/8/31 | <.001 | 2983 | 1447.89 | 2.06 | 2023 | 621.99 |

**Table S3. Annual global spatial autocorrelation (Moran’s I)**

| Year | Moran’s I | Z score | P value |
| --- | --- | --- | --- |
| 2008 | 0.43 | 3.49 | <.001 |
| 2009 | -0.27 | -1.29 | .90 |
| 2010 | 0.01 | 0.64 | .26 |
| 2011 | -0.21 | -0.96 | .83 |
| 2012 | 0.11 | 1.26 | .10 |
| 2013 | -0.01 | 0.41 | .34 |
| 2014 | -0.16 | -0.63 | .74 |
| 2015 | -0.06 | 0.02 | .49 |
| 2016 | 0.38 | 2.87 | .002 |
| 2017 | 0.05 | 0.71 | .24 |
| 2018 | -0.07 | -0.03 | .51 |
| 2019 | 0.14 | 1.36 | .09 |
| 2020 | 0.12 | 1.42 | .08 |
| 2021 | 0.26 | 2.16 | .02 |
| 2022 | 0.01 | 0.50 | .31 |
| 2023 | 0.01 | 0.54 | .30 |

**Table S4. Annual observed versus counterfactual totals and 95% prediction intervals for ITS models**

| Model | Period | Year | Actual cases | Predicted cases | Lower 95% PI | Upper 95% PI | Difference | Percent change (%) |
| --- | --- | --- | --- | --- | --- | --- | --- | --- |
| All reported HFMD (Vaccine-era) | 2017-2019 | 2017 | 80344 | 104712 | 84388 | 129080 | -24368 | -23.27 |
| All reported HFMD (Vaccine-era) | 2017-2019 | 2018 | 115029 | 103091 | 82975 | 130561 | 11938 | 11.58 |
| All reported HFMD (Vaccine-era) | 2017-2019 | 2019 | 70759 | 101494 | 82487 | 132654 | -30735 | -30.28 |
| Typed EV71 (Vaccine-era) | 2017-2019 | 2017 | 470 | 659 | 467 | 933 | -189 | -28.70 |
| Typed EV71 (Vaccine-era) | 2017-2019 | 2018 | 181 | 531 | 379 | 798 | -350 | -65.89 |
| Typed EV71 (Vaccine-era) | 2017-2019 | 2019 | 63 | 427 | 305 | 662 | -364 | -85.25 |
| Typed CV-A16 (Vaccine-era) | 2017-2019 | 2017 | 670 | 2411 | 1584 | 3969 | -1741 | -72.21 |
| Typed CV-A16 (Vaccine-era) | 2017-2019 | 2018 | 999 | 3858 | 2544 | 6932 | -2859 | -74.11 |
| Typed CV-A16 (Vaccine-era) | 2017-2019 | 2019 | 1653 | 6174 | 4001 | 12183 | -4521 | -73.23 |
| Typed other enteroviruses (Vaccine-era) | 2017-2019 | 2017 | 1745 | 1980 | 1633 | 2437 | -235 | -11.88 |
| Typed other enteroviruses (Vaccine-era) | 2017-2019 | 2018 | 2932 | 2431 | 2010 | 3111 | 501 | 20.59 |
| Typed other enteroviruses (Vaccine-era) | 2017-2019 | 2019 | 2203 | 2985 | 2461 | 3883 | -782 | -26.20 |
| All reported HFMD (COVID-19-period) | 2020-2023 | 2020 | 52712 | 149675 | 96603 | 222919 | -96963 | -64.78 |
| All reported HFMD (COVID-19-period) | 2020-2023 | 2021 | 53122 | 162121 | 105863 | 243423 | -108999 | -67.23 |
| All reported HFMD (COVID-19-period) | 2020-2023 | 2022 | 28385 | 175601 | 111465 | 268202 | -147216 | -83.84 |
| All reported HFMD (COVID-19-period) | 2020-2023 | 2023 | 70288 | 190201 | 127746 | 284820 | -119913 | -63.05 |
| Typed EV71 (COVID-19-period) | 2020-2023 | 2020 | 87 | 395 | 158 | 871 | -308 | -77.96 |
| Typed EV71 (COVID-19-period) | 2020-2023 | 2021 | 72 | 355 | 137 | 846 | -283 | -79.72 |
| Typed EV71 (COVID-19-period) | 2020-2023 | 2022 | 106 | 319 | 129 | 764 | -213 | -66.81 |
| Typed EV71 (COVID-19-period) | 2020-2023 | 2023 | 313 | 287 | 113 | 678 | 26 | 8.93 |
| Typed CV-A16 (COVID-19-period) | 2020-2023 | 2020 | 220 | 1992 | 1061 | 3568 | -1772 | -88.96 |
| Typed CV-A16 (COVID-19-period) | 2020-2023 | 2021 | 1123 | 2406 | 1239 | 4598 | -1283 | -53.33 |
| Typed CV-A16 (COVID-19-period) | 2020-2023 | 2022 | 776 | 2907 | 1597 | 5228 | -2131 | -73.30 |
| Typed CV-A16 (COVID-19-period) | 2020-2023 | 2023 | 385 | 3511 | 1848 | 6474 | -3126 | -89.03 |
| Typed other enteroviruses (COVID-19-period) | 2020-2023 | 2020 | 4122 | 5512 | 3482 | 8156 | -1390 | -25.22 |
| Typed other enteroviruses (COVID-19-period) | 2020-2023 | 2021 | 3128 | 7507 | 4864 | 11503 | -4379 | -58.33 |
| Typed other enteroviruses (COVID-19-period) | 2020-2023 | 2022 | 1412 | 10224 | 6604 | 15851 | -8812 | -86.19 |
| Typed other enteroviruses (COVID-19-period) | 2020-2023 | 2023 | 5186 | 13924 | 9215 | 21396 | -8738 | -62.76 |

**Table S5. Annual reported HFMD cases, typed cases, typing fractions, and typed serotype composition in Anhui Province, 2008-2023**

| Year | Reported cases | Records with serotype field | Typed cases | EV71 typed cases | CV-A16 typed cases | Other typed cases | Typed fraction (%) | EV71 share (%) | CV-A16 share (%) | Other share (%) |
| --- | --- | --- | --- | --- | --- | --- | --- | --- | --- | --- |
| 2008 | 26359 | 76 | 76 | 69 | 0 | 7 | 0.29 | 90.79 | 0.00 | 9.21 |
| 2009 | 55925 | 500 | 500 | 251 | 83 | 166 | 0.89 | 50.20 | 16.60 | 33.20 |
| 2010 | 84323 | 2229 | 2229 | 1166 | 681 | 382 | 2.64 | 52.31 | 30.55 | 17.14 |
| 2011 | 82739 | 2403 | 2403 | 1634 | 398 | 371 | 2.90 | 68.00 | 16.56 | 15.44 |
| 2012 | 105554 | 2542 | 2542 | 1059 | 870 | 613 | 2.41 | 41.66 | 34.23 | 24.11 |
| 2013 | 90447 | 2692 | 2692 | 1497 | 177 | 1018 | 2.98 | 55.61 | 6.58 | 37.82 |
| 2014 | 145528 | 3268 | 3268 | 1405 | 1092 | 771 | 2.25 | 42.99 | 33.41 | 23.59 |
| 2015 | 92572 | 2770 | 2770 | 532 | 763 | 1475 | 2.99 | 19.21 | 27.55 | 53.25 |
| 2016 | 138493 | 3664 | 3664 | 1266 | 900 | 1498 | 2.65 | 34.55 | 24.56 | 40.88 |
| 2017 | 80344 | 2885 | 2885 | 470 | 670 | 1745 | 3.59 | 16.29 | 23.22 | 60.49 |
| 2018 | 115029 | 4112 | 4112 | 181 | 999 | 2932 | 3.58 | 4.40 | 24.29 | 71.30 |
| 2019 | 70759 | 3919 | 3919 | 63 | 1653 | 2203 | 5.54 | 1.61 | 42.18 | 56.21 |
| 2020 | 52712 | 4429 | 4429 | 87 | 220 | 4122 | 8.40 | 1.96 | 4.97 | 93.07 |
| 2021 | 53122 | 4323 | 4323 | 72 | 1123 | 3128 | 8.14 | 1.67 | 25.98 | 72.36 |
| 2022 | 28385 | 2294 | 2294 | 106 | 776 | 1412 | 8.08 | 4.62 | 33.83 | 61.55 |
| 2023 | 70288 | 5884 | 5884 | 313 | 385 | 5186 | 8.37 | 5.32 | 6.54 | 88.14 |

**Table S6. Annual severe-proportion summaries from the exploratory severe-case ITS models**

| Scenario | Period | Year | Actual severe cases | Actual total cases | Observed severe proportion | Expected severe proportion | Expected proportion LCL | Expected proportion UCL |
| --- | --- | --- | --- | --- | --- | --- | --- | --- |
| EV71 vaccine | 2017-2019 | 2017 | 155 | 80344 | 0.001929 | 0.009499 | 0.007295 | 0.012386 |
| EV71 vaccine | 2017-2019 | 2018 | 69 | 115029 | 0.000600 | 0.020809 | 0.014483 | 0.029864 |
| EV71 vaccine | 2017-2019 | 2019 | 10 | 70759 | 0.000141 | 0.053469 | 0.033073 | 0.085335 |
| COVID-19 | 2020-2023 | 2020 | 3 | 52712 | 0.000057 | 0.001298 | 0.001102 | 0.001531 |
| COVID-19 | 2020-2023 | 2021 | 8 | 53122 | 0.000151 | 0.001167 | 0.000985 | 0.001386 |
| COVID-19 | 2020-2023 | 2022 | 3 | 28385 | 0.000106 | 0.001050 | 0.000880 | 0.001255 |
| COVID-19 | 2020-2023 | 2023 | 11 | 70288 | 0.000156 | 0.000944 | 0.000785 | 0.001137 |

**Table S7. Annual severe cases, deaths, and case-fatality summaries**

| Year | Reported cases | Severe cases | Deaths | Deaths per 100,000 reported cases |
| --- | --- | --- | --- | --- |
| 2008 | 26359 | 33 | 26 | 98.64 |
| 2009 | 55925 | 72 | 3 | 5.36 |
| 2010 | 84323 | 584 | 20 | 23.72 |
| 2011 | 82739 | 432 | 15 | 18.13 |
| 2012 | 105554 | 250 | 5 | 4.74 |
| 2013 | 90447 | 232 | 19 | 21.01 |
| 2014 | 145528 | 451 | 22 | 15.12 |
| 2015 | 92572 | 179 | 3 | 3.24 |
| 2016 | 138493 | 572 | 5 | 3.61 |
| 2017 | 80344 | 155 | 3 | 3.73 |
| 2018 | 115029 | 69 | 1 | 0.87 |
| 2019 | 70759 | 10 | 1 | 1.41 |
| 2020 | 52712 | 3 | 0 | 0.00 |
| 2021 | 53122 | 8 | 0 | 0.00 |
| 2022 | 28385 | 3 | 0 | 0.00 |
| 2023 | 70288 | 11 | 0 | 0.00 |
| 2008-2023 | 1292579 | 3064 | 123 | 9.52 |

Note: Deaths were identified from nonmissing death dates in the surveillance extract. Follow-up information on neurological sequelae was not available in the extract.

**Table S8. Multinomial regression of typed serotype composition adjusted for year, sex, age group, and severity**

| Outcome | Term | Estimate | Standard error | RR | RR LCL | RR UCL |
| --- | --- | --- | --- | --- | --- | --- |
| EV71 | Intercept | 1.93 | 0.04 | 6.89 | 6.33 | 7.50 |
| EV71 | Year | -0.40 | 0.00 | 0.67 | 0.66 | 0.67 |
| EV71 | Male  (vs female) | 0.01 | 0.03 | 1.01 | 0.95 | 1.07 |
| EV71 | Age 2-3 years  (vs <=1 year) | 0.35 | 0.03 | 1.41 | 1.33 | 1.50 |
| EV71 | Age 4+ years  (vs <=1 year) | 0.73 | 0.04 | 2.08 | 1.93 | 2.24 |
| EV71 | Severe  (vs mild) | 1.96 | 0.08 | 7.08 | 6.01 | 8.34 |
| CV-A16 | Intercept | 0.39 | 0.04 | 1.48 | 1.37 | 1.61 |
| CV-A16 | Year | -0.18 | 0.00 | 0.84 | 0.83 | 0.84 |
| CV-A16 | Male  (vs female) | -0.05 | 0.02 | 0.95 | 0.90 | 0.99 |
| CV-A16 | Age 2-3 years  (vs <=1 year) | 0.51 | 0.03 | 1.66 | 1.57 | 1.75 |
| CV-A16 | Age 4+ years  (vs <=1 year) | 0.97 | 0.03 | 2.64 | 2.48 | 2.81 |
| CV-A16 | Severe  (vs mild) | -0.69 | 0.14 | 0.50 | 0.38 | 0.67 |

**Table S9. Vaccine-era training-window sensitivity for ITS counterfactual summaries**

| Endpoint and model | Year | Actual cases | Predicted cases | Lower 95% PI | Upper 95% PI | Difference | Percent change (%) |
| --- | --- | --- | --- | --- | --- | --- | --- |
| All reported HFMD - Primary 2013-2016 linear | 2017 | 80344 | 104712 | 84388 | 129080 | -24368 | -23.27 |
| All reported HFMD - Primary 2013-2016 linear | 2018 | 115029 | 103091 | 82975 | 130561 | 11938 | 11.58 |
| All reported HFMD - Primary 2013-2016 linear | 2019 | 70759 | 101494 | 82487 | 132654 | -30735 | -30.28 |
| All reported HFMD - Sensitivity 2010-2016 quadratic | 2017 | 80344 | 86241 | 65710 | 116835 | -5897 | -6.84 |
| All reported HFMD - Sensitivity 2010-2016 quadratic | 2018 | 115029 | 68600 | 52548 | 101386 | 46429 | 67.68 |
| All reported HFMD - Sensitivity 2010-2016 quadratic | 2019 | 70759 | 51198 | 37663 | 85644 | 19561 | 38.21 |
| Typed EV71 - Primary 2013-2016 linear | 2017 | 470 | 659 | 467 | 933 | -189 | -28.70 |
| Typed EV71 - Primary 2013-2016 linear | 2018 | 181 | 531 | 379 | 798 | -350 | -65.89 |
| Typed EV71 - Primary 2013-2016 linear | 2019 | 63 | 427 | 305 | 662 | -364 | -85.25 |
| Typed EV71 - Sensitivity 2010-2016 quadratic | 2017 | 470 | 566 | 386 | 899 | -96 | -17.01 |
| Typed EV71 - Sensitivity 2010-2016 quadratic | 2018 | 181 | 360 | 241 | 678 | -179 | -49.74 |
| Typed EV71 - Sensitivity 2010-2016 quadratic | 2019 | 63 | 210 | 136 | 467 | -147 | -69.96 |
| Typed CV-A16 - Primary 2013-2016 linear | 2017 | 670 | 2411 | 1584 | 3969 | -1741 | -72.21 |
| Typed CV-A16 - Primary 2013-2016 linear | 2018 | 999 | 3858 | 2544 | 6932 | -2859 | -74.11 |
| Typed CV-A16 - Primary 2013-2016 linear | 2019 | 1653 | 6174 | 4001 | 12183 | -4521 | -73.23 |
| Typed CV-A16 - Sensitivity 2010-2016 quadratic | 2017 | 670 | 1321 | 842 | 2269 | -651 | -49.28 |
| Typed CV-A16 - Sensitivity 2010-2016 quadratic | 2018 | 999 | 1667 | 1058 | 3827 | -668 | -40.06 |
| Typed CV-A16 - Sensitivity 2010-2016 quadratic | 2019 | 1653 | 2155 | 1317 | 6800 | -502 | -23.30 |
| Typed other enteroviruses - Primary 2013-2016 linear | 2017 | 1745 | 1980 | 1633 | 2437 | -235 | -11.88 |
| Typed other enteroviruses - Primary 2013-2016 linear | 2018 | 2932 | 2431 | 2010 | 3111 | 501 | 20.59 |
| Typed other enteroviruses - Primary 2013-2016 linear | 2019 | 2203 | 2985 | 2461 | 3883 | -782 | -26.20 |
| Typed other enteroviruses - Sensitivity 2010-2016 quadratic | 2017 | 1745 | 2161 | 1701 | 2957 | -416 | -19.24 |
| Typed other enteroviruses - Sensitivity 2010-2016 quadratic | 2018 | 2932 | 2603 | 1979 | 3928 | 329 | 12.63 |
| Typed other enteroviruses - Sensitivity 2010-2016 quadratic | 2019 | 2203 | 3068 | 2302 | 5335 | -865 | -28.20 |

**Table S10. COVID-19-period sensitivity using a pre-vaccine baseline for ITS counterfactual summaries**

| Endpoint and model | Year | Actual cases | Predicted cases | Lower 95% PI | Upper 95% PI | Difference | Percent change (%) |
| --- | --- | --- | --- | --- | --- | --- | --- |
| All reported HFMD - Pre-vaccine 2008-2015 linear | 2020 | 52712 | 445038 | 237865 | 832655 | -392326 | -88.16 |
| All reported HFMD - Pre-vaccine 2008-2015 linear | 2021 | 53122 | 543871 | 279182 | 1059509 | -490749 | -90.23 |
| All reported HFMD - Pre-vaccine 2008-2015 linear | 2022 | 28385 | 664653 | 327089 | 1350590 | -636268 | -95.73 |
| All reported HFMD - Pre-vaccine 2008-2015 linear | 2023 | 70288 | 812257 | 382645 | 1724213 | -741969 | -91.35 |
| Typed EV71 - Pre-vaccine 2008-2015 linear | 2020 | 87 | 1106 | 469 | 2608 | -1019 | -92.13 |
| Typed EV71 - Pre-vaccine 2008-2015 linear | 2021 | 72 | 1856 | 744 | 4632 | -1784 | -96.12 |
| Typed EV71 - Pre-vaccine 2008-2015 linear | 2022 | 106 | 1984 | 743 | 5295 | -1878 | -94.66 |
| Typed EV71 - Pre-vaccine 2008-2015 linear | 2023 | 313 | 2120 | 741 | 6066 | -1807 | -85.24 |
| Typed CV-A16 - Pre-vaccine 2008-2015 linear | 2020 | 220 | 2050 | 905 | 4644 | -1830 | -89.27 |
| Typed CV-A16 - Pre-vaccine 2008-2015 linear | 2021 | 1123 | 3514 | 1466 | 8423 | -2391 | -68.04 |
| Typed CV-A16 - Pre-vaccine 2008-2015 linear | 2022 | 776 | 4347 | 1702 | 11102 | -3571 | -82.15 |
| Typed CV-A16 - Pre-vaccine 2008-2015 linear | 2023 | 385 | 5180 | 1900 | 14123 | -4795 | -92.57 |
| Typed other enteroviruses - Pre-vaccine 2008-2015 linear | 2020 | 4122 | 10903 | 6416 | 18527 | -6781 | -62.19 |
| Typed other enteroviruses - Pre-vaccine 2008-2015 linear | 2021 | 3128 | 15814 | 8951 | 27938 | -12686 | -80.22 |
| Typed other enteroviruses - Pre-vaccine 2008-2015 linear | 2022 | 1412 | 22937 | 12468 | 42199 | -21525 | -93.84 |
| Typed other enteroviruses - Pre-vaccine 2008-2015 linear | 2023 | 5186 | 33269 | 17342 | 63828 | -28083 | -84.41 |

Note: This sensitivity analysis trained the COVID-19 counterfactual only on January 2008 to December 2015, before EV71 vaccine introduction, and projected it forward to 2020-2023 so that the COVID baseline did not include vaccine-era months.
